# Student-performed periodontal therapy: retrospective cohort study on outcomes and related recommendations for enhancing undergraduate periodontal education

**DOI:** 10.1186/s12909-025-07699-2

**Published:** 2025-07-31

**Authors:** Marco M. Herz, Judith Schamuhn, Boris Krumm, Valentin Bartha

**Affiliations:** 1 Department for Conservative Dentistry, University of Tübingen, University Hospital, Osianderstr. 2-8, 72076 Tuebingen, Germany; 2 Private Practice, Cologne, Germany; 3 Private Practice, Franfurt/Main, Germany; 4 Department for Conservative Dentistry, Heidelberg University, Im Neuenheimer Feld 400, 69120 Heidelberg, Germany

**Keywords:** Periodontics / education, Dental schools, Dental students, Periodontitis, Periodontal treatment, Supportive periodontal care

## Abstract

**Background:**

The aim of the study was to evaluate periodontal treatment success in patients treated by undergraduates. The findings shall be used to gain implications for future curriculum frameworks.

**Methods:**

In this retrospective cohort study, the medical records of 107 patients (median age 58 years, 48% female) diagnosed with stage III periodontitis who were treated by students were analyzed. Anamnestic and periodontal clinical parameters (bleeding on probing (BOP%), periodontal pocket depths (PD), plaque control record (PCR) were extracted at baseline (T0), re-evaluation (T1, median 3mth), and last SPC visit (T2, median 47mth after T0). The primary outcome was assessing the relative proportion of patients achieving specific treatment endpoints at T1 (EP1 = BOP < 20% and EP2 = ≤ 4site with PD ≥ 5 mm), secondary outcomes included identification of factors influencing these specific endpoints at T1 and T2.

**Results:**

All parameters (BOP, PD, PCR) improved statistically significantly during step 1, 2 and 4 of periodontal therapy. In contrast, a worsening of PCR between T1 and T2 (*p* = 0.049) was observed. Achieving EP1 at T1 was achieved by 49% of al patients. It was positively affected by being female and negatively by T0 BOP (OR3.90, *p* = 0.008, OR0.95, *p* < 0.001), EP2 at T1 was achieved by 29% of all patients and influenced by the number of PD ≥ 6 mm at T0 (OR0.98, *p* < 0.001). At T2 the relative proportion of patients dropped to 37%(EP1) and 26%(EP2), with EP1 significantly affected by plaque control at T2 (OR0.96, *p* = 0.003) and EP2 significantly affected by PD ≥ 6 mm at T1 (OR0.69, *p* < 0.001).

**Conclusions:**

Non-surgical therapy performed by students significantly improves periodontal outcomes in Stage III periodontitis. Particularly residual pockets and plaque seemed to affect unmet treatment targets.

**Supplementary Information:**

The online version contains supplementary material available at 10.1186/s12909-025-07699-2.

## Introduction

Periodontitis is a chronic, multifactorial inflammatory disease associated with dysbiotic plaque biofilms, leading to the progressive destruction of the periodontium. Typical features include tissue loss, clinically measurable attachment loss (CAL), radiographically visible bone loss, periodontal pockets, and gingival bleeding [1]. Periodontology is a key and complex field in dentistry, particularly due to its connection with general medicine [2–4]. New findings on its aetiology and pathogenesis relate to nutrition, lifestyle, and psychological stress [5–8].

Treatment requires thorough supra- and subgingival removal of biofilm and calculus by dental professionals, along with continuous domestic plaque control [9]. Instruments used include curettes, ultrasonic and sonic scalers, and air-polishing devices [10]. In severe cases, antibiotics and antiseptics such as chlorhexidine or povidone-iodine are used as adjuncts [11–16].

Long-term treatment success can be challenging due to risk factors can influence success like smoking, diabetes, poor patient compliance, and anatomical conditions that hinder effective subgingival cleaning [2, 17–22].

In 2020, Feres et al. defined criteria for long-term treatment success [23] (e.g. pocket depth/PD ≥ 6 mm as a risk factor) and highlighted the need for surgical intervention in cases with residual deep pockets [16, 24].

In many countries, students are required to perform standard dental procedures under supervision, including periodontal treatment. The European Federation of Periodontology (EFP) mandates knowledge of periodontal-systemic interactions and clinical competence in diagnosing and managing periodontal diseases [25]. According to the German National Competence-Based Catalogue of Learning Objectives for Dentistry (NKLZ), graduates must be able to justify and, if necessary, perform periodontal therapy [26]. National licensing regulations also require practical examinations on patients [27].

Dental education in Germany—and likely in other countries—faces structural disparities: some universities have dedicated periodontology departments; others lack specialised training. These differences have a direct impact on students’ ability to manage complex treatment cases—particularly in light of the new and technically more demanding methods for subgingival debridement [28], as well as the increasing and thus more extensive need for periodontal treatments with more severe forms in senior citizens due to demographic change [29, 30]. Rigid semester schedules also conflict with individualised supportive periodontal care (SPC) intervals, risking suboptimal long-term results [17]. Several studies have addressed diagnostic and planning training [31–34] but few have investigated student-performed treatments—none analyzing factors influencing success [35–38]. Alvarez-Azaustre et al. found that even non-surgical periodontal treatment by students improves patients’ health-related quality of life [39]. Figuero et al. defined the content of periodontal education in their 19th Workshop of Periodontology, placing particular emphasis on Steps 1 and 2 of periodontal treatment [40]. While Step 3 must be recognized and explained to the patient, its execution should be carried out by experienced periodontists or dentists via referral. This approach appears appropriate in the context of technically demanding and aesthetic considerations. The findings of the DMS 6 study suggest a projected increase in the prevalence of severe periodontitis, accompanied by a trend toward longer tooth retention among patients in the future [40]. Whether the number of periodontally specialized dentists will, in contrast to the current situation, be available in sufficient numbers—extrapolated to the German population and within reasonable proximity to dental practices—remains debatable. While postgraduate training is fortunately on a promising trajectory, with recent positive developments such as the introduction of a new regional postgraduate periodontist training and the accreditation of the EFP-specialist program in Frankfurt, it remains uncertain whether these measures will be sufficient to meet future demands. Postgraduate Masterprograms in Periodontology might further contribute to a broader availability of dentists with advanced periodontal skills. Nevertheless, a more robust, yet accessible, undergraduate education including periodontal surgery appears to be a sensible step forward.

This retrospective study evaluated the outcomes of non-surgical periodontal therapy performed exclusively by students. The primary endpoint was the frequency of achieving the target values (maximum of 4 sites with PD ≥ 5 mm) defined by Feres et al. [23].

While BOP thresholds of ≤ 10% or ≤ 20% were additionally discussed by Feres et al., they were not part of their primary endpoint. However, in this study, we separately evaluated beside the proportion of patients reaching the PD-based treat-to-target endpoint also those achieving a BOP ≤ 20%, to provide a comprehensive assessment of treatment response and potential implications for undergraduate education. As secondary outcomes we assessed factors influencing these specific endpoints at T1 and T2.

## Materials and methods

### Study design

**Fig. 1:**
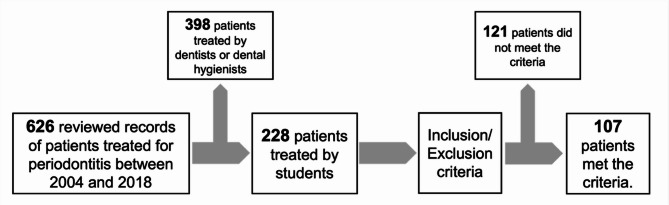
Process of patient record selection

This was a retrospective cohort study based on the medical records of patients who underwent periodontal treatment performed by students between 2004 and 2018. The patients were initially identified by an electronic database search in the Department of Conservative Dentistry at the University Hospital of Tuebingen and then selected based on the data in their patient files (Fig. 1). The patient records were analysed for the complete documentation:

- periodontal pocket depth (PD), at six sites per tooth,
- bleeding on probing (BOP),
- furcation involvement (FI) [41],
- tooth mobility (TM) [42],
- and plaque control record (PCR) [43];

### Inclusion criteria

- At T0 (baseline).
  - Panoramic radiographs or a survey of periapical radiographs ≤ 12 months.
  - Participants age ≥ 18 years.
  - Diagnosis of periodontitis stage III.
- A complete dental and periodontal examination, which includes PD, BOP, and PCR at the start of therapy (T0), after Steps 1 and 2 of active periodontal therapy (re-evaluation, T1), and during SPC (last documented visit, T2).

### Exclusion criteria

- Incomplete documentation of periodontal parameters.

This study analyzed data from 73 of the 107 participants previously included in Bartha et al. (2022), with 56 of them also considered in the results of Herz et al. (2024) and (2025) [44–46].

### Student courses and applied treatment concept

At the dental school in Tuebingen, periodontitis patients are treated in Courses I and II of the Department of Conservative Dentistry, with up to 31 students per course. All treatment steps are supervised and, if necessary, adjusted by licensed dentists or periodontists. Patients come to the clinic independently or are referred by practitioners or other departments (e.g. prosthetics) for required periodontal treatment under statutory health insurance.

Once the need is confirmed, patients are informed about student treatment, including its benefits (e.g., cost savings) and drawbacks (e.g., longer and more frequent appointments and are only enrolled if they explicitly agree. During the entire observation period, treatments followed a consistent departmental protocol in terms of structure, procedures, and documentation. Periodontal parameters were recorded using a standardized, institution-wide charting form. All treatments were performed under direct supervision by licensed teaching staff.

**Fig. 2:**
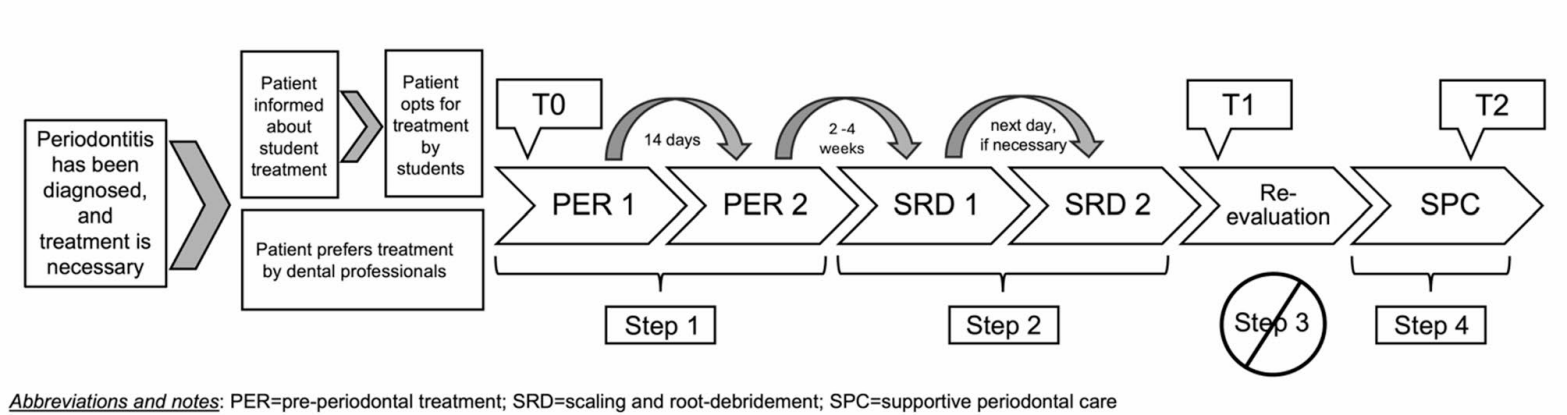
Procedure for standardized periodontitis treatment at the dental clinic

Lectures covered only the classical risk factors such as diabetes and smoking. Recently emphasized aspects in the literature—such as nutrition, stress, or body weight—were not included in the theoretical curriculum. This was also reflected in practical student treatment. Patient anamnesis was limited to asking whether the individual smoked or had diabetes, while complementary measures—such as recommending a smoking cessation program, nutritional counseling, stress management, or weight reduction—were neither explicitly addressed nor part of the treatment concept. Figure 2 shows the general workflow of periodontal treatment in the Department of Conservative Dentistry. For a detailed description of the applied treatment concept see Appendix 1.

### Supportive antibiotic treatment

At that time, additional antibiotics were administered during Step 2 or T1 at the discretion of the course supervisor if periodontitis was severe or diagnosed as aggressive or severe chronic [47, 48]. The IAI-Pado DNA Test 4.5 (Institute for Applied Immunology in Zuchwil, Switzerland) was routinely used to identify pathogenic oral microbes. Depending on the case, amoxicillin, metronidazole, their combination, or clindamycin/tetracycline (in case of allergy) were prescribed [48].

### Patient charts

The periodontal examination charts for T0, T1 and T2 (last documented visit) were evaluated.

For each patient, the following data were documented:

- PD (mm) at six sites per tooth.
- BOP (%) and PCR (%).
- Proportion/number of teeth with PD ≤ 4 mm, PD = 5 mm, and PD ≥ 6 mm.

Baseline radiographs were evaluated to visually assess maximum interdental bone loss. Charts were also reviewed for smoking status (non-smokers, former smokers who quit ≥ 5 years ago, and smokers). In deviation from the classification of smoking behavior as proposed by Lang and Tonetti—non-smokers, former smokers who quit ≥ 5 years ago, smokers ≤ 10 cig./day, > 10 cig./day, and ≥ 20 cig./day—no differentiation was made among smokers based on the number of cigarettes consumed per day, as required by the classification [17]. Instead, all smokers were assigned to Grade B, although a consumption of ≥ 10 cigarettes per day would have justified classification as Grade C. The presence of diabetes mellitus was also reviewed and documented.

Where applicable, and where the necessary data were duly documented in the patient records, the recommendations of ACES [49] were followed: Initial periodontal diagnoses were reclassified using the current classification system based on the available data. This included maximum baseline PD, bone loss, diabetes, smoking status and and—where clearly identifiable—periodontal tooth loss (PTL). The grade was determined using the bone loss age index and upgraded if diabetes and/or smoking were present. The following information and data documented in the patient files were used to answer the research questions:

- Characteristic patient data: sex, age, smoking status and presence of diabetes mellitus. In the case of the latter, it should be noted that the HbA1c value (glycated hemoglobin) was often not documented and was therefore not used.
- Periodontal data: PCR, bone loss/BL, PD and BOP.
- Treatment data: Number of SPC appointments, duration of therapy steps, use of local anaesthesia during scaling and the use of antibiotics.

### Retrospective power analysis

As no a priori sample size calculation was performed a post hoc power analysis was conducted to evaluate whether the available sample size (*n* = 107) was sufficient to detect an association between the number of sites with PD ≥ 6 mm at baseline (T0) and clinical treatment success as defined by Feres et al. (≤ 4 sites with PD ≥ 5 mm at T1). Logistic regression analysis revealed a statistically significant inverse association (OR = 0.85, 95% CI: 0.78–0.92, *p* < 0.001). Based on the narrow confidence interval and low p-value, the statistical power for detecting this effect was deemed sufficient.

### Statistical analysis

The patient was considered a statistical unit. The primary outcome “clinical treatment success” followed thresholds by Feres et al. (2020): BOP ≤ 20% and ≤ 4 sites with PD ≥ 5 mm. Notably, we differentiated between the treat-to-target endpoint (≤ 4 sites with PD ≥ 5 mm) and the supplementary BOP ≤ 20% threshold, which, though discussed by Feres et al., was not part of their formal definition. All other parameters were secondary outcomes.

Descriptive data were reported as absolute/relative frequencies, means ± SDs, and medians with interquartile ranges. Normal distribution was tested using the Anderson–Darling test. As variables were not normally distributed, time points were compared via the Wilcoxon rank-sum test. Nominal regression identified factors influencing residual pockets with STN at T1 and T2, and clinical endpoints per Feres et al. (2020). Potential confounders for outcomes at T1 were first analyzed univariately: BOPT0, BOPT1, PDT0 ≥ 6 mm (n), PCRT0, PCRT1, sex, age, smoking status, type 2 diabetes mellitus (DMT2), grade (A-C), systemic antibiotics, and local anaesthesia in Step 2. For T2 outcomes, the following were tested: BOPT1, BOPT2, PDT1 ≥ 6 mm (n), PCRT1, PCRT2, sex, age, smoking status, DMT2, SPC/y ratio (conducted vs. recommended), and ≥ 2 SPC/y. Significant variables were included in multivariate models: Model 1 (age, sex) and Model 2 (all variables). A 0.05 significance level was applied. Analyses were conducted using JMP software (SAS JMP 16.0, SAS Institute GmbH, Germany).

## Results

A total of 107 patients (27–84 years, median age 58 years, Q1-Q3: 50–65 years) were included in the statistical analysis for the baseline (Table 1), of whom 48% were female. Most patients were classified as grade B (72%). The median duration of T0 to T1 was 3 months (Q1-Q3: 2–6) and that of T1 to T2 was 47 months (Q1-Q3: 42–49). The mean annual visits for SPC in the overall cohort was 1.67 ± 0.42 (Table 1).

**Table 1:** Baseline characteristics of the participants

Patients	Total	Male	Female	Smoker	Diabetes mellitus	Periodontitis Grading		
						Grade A	Grade B	Grade C
n	107	56	51	33	14	4	70	33
%	100%	52%	48%	31%	13%	4%	72%	24%

All clinical parameters improved significantly between T0 and T1, as well as between T0 and T2. However, the changes between T1 and T2 were mostly not statistically significant, only the PCR deteriorated statistically significantly (*p* < 0.001); see Table 2.

**Table 2:** Clinical parameter development at T0, T1, and T2

T0	Mean	SD	Median	IQR	*p*-value	
PD ≤ 4 mm (%)	83	15	87	77–94		
PD = 5 mm (n / %)	9.85 / 7	6.99 / 5	8.00 / 6	4.00–15 / 3–10		
PD ≥ 6 mm (n / %)	13.34 / 10	18.06 / 12	9.00 / 6	3.00–17 / 2–13		
BOP (%)	41	26	36	20–57		
PCR (%)	54	19	53	38–68		
**T1**					**T0-T1**	
PD ≤ 4 mm (%)	90	10	93	87–98		
PD = 5 mm (n / %)	6.36 / 5	5.87 / 5	5.00 / 4	2.00–9.00 / 2–7	< 0.001*	
PD ≥ 6 mm (n / %)	5.44 / 5	6.25 / 6	3.00 / 3	1.00–8.00 / 1–7	< 0.001*	
BOP (%)	27	22	21	10–37	< 0.001*	
PCR (%)	40	22	40	23–55	< 0.001*	
**T2**					**T0-T2**	**T1-T2**
PD ≤ 4 mm (%)	89	12	93	85–97		
PD = 5 mm (n / %)	7.69 / 6	7.67 / 6	6.00 / 5	2.00–10.00 / 2–8	0.011*	0.177
PD ≥ 6 mm (n / %)	6.44 / 5	9.11 / 8	3.00 / 2	1.00–8.00 / 1–6	< 0.001*	0.868
BOP (%)	27	17	23	13–37	< 0.001*	0.433
PCR (%)	49	20	50	35–63	0.049*	< 0.001*

*Abbreviations and notes*: T0 = baseline; T1 = re-evaluation; T2 = last recorded SPC appointment; SD = standard deviation; IQR = interquartilrange; PD = pocket depth; BOP = bleeding on probing; PCR = plaque control record; All p-values were calculated using the Wilcoxon signed-rank test; * statistically significant

### Residual pockets with surgical treatment need after Step 2 of periodontal therapy at T1 and during Step 4 at T2

**Fig. 3:**
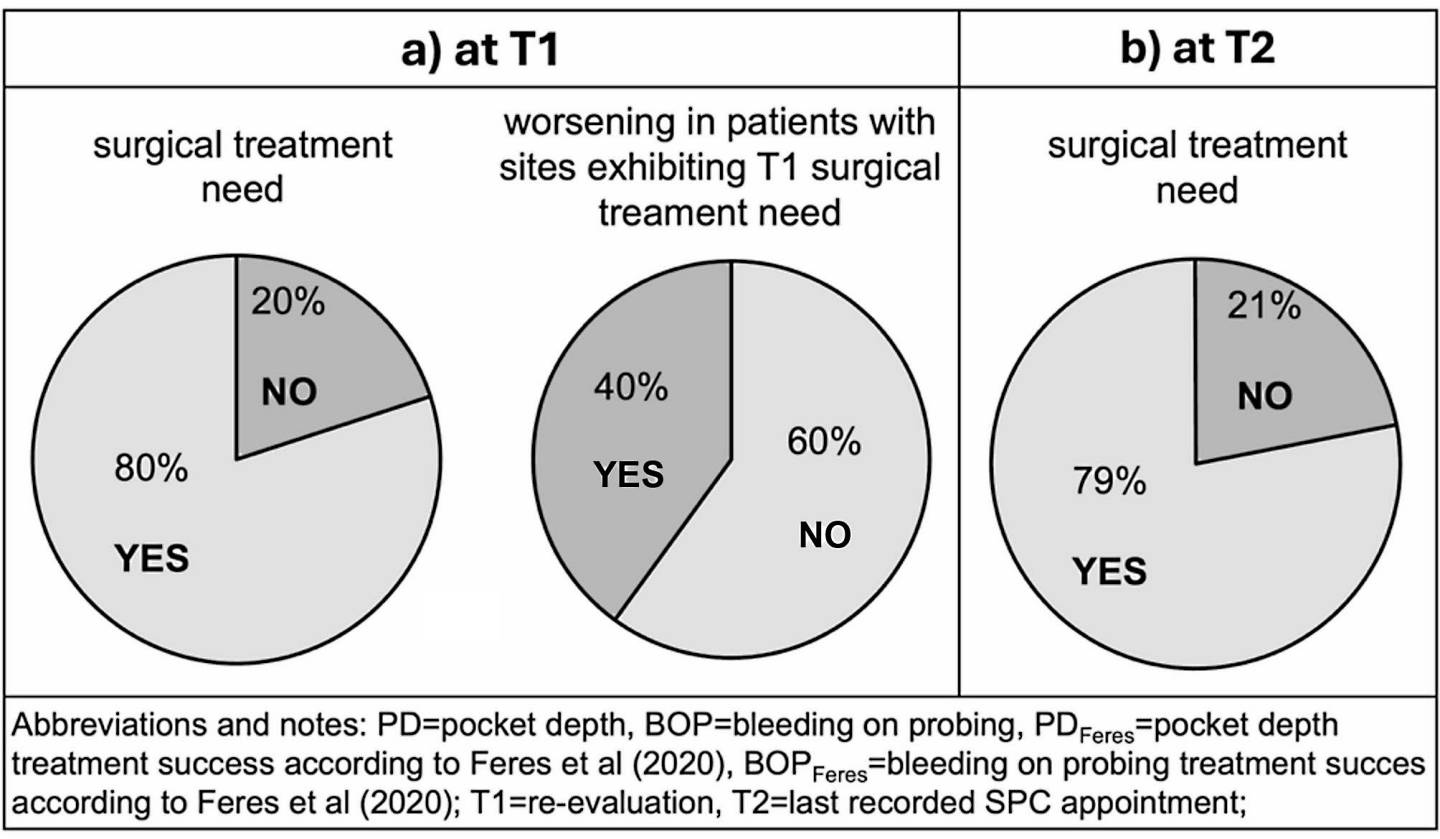
Relative number of patients with STN at T1 and T2 and relative number of patients with worsening, dependent of STN at T1

After Step 2 of periodontal therapy, 80% of all patients showed at least one site with STN. Moreover, the 40% of patients with surgical treatment need at T1 showed an increasing number of sites with PD ≥ 6 mm during Step 4 (Fig. 3a). At T2, 79% of all patients showed at least one site with a STN (Fig. 3b).

### Evaluation of treatment success according to the framework of Feres et al. (2020)

**Fig. 4:**
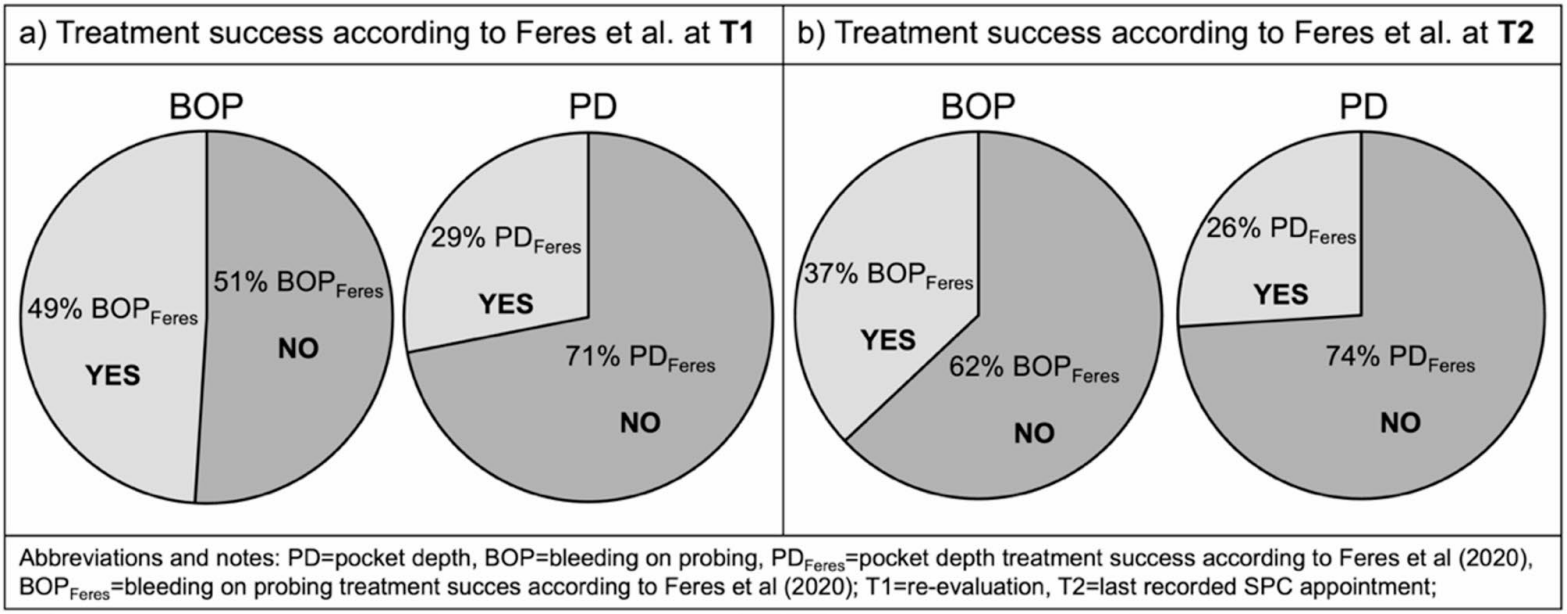
Relative number of patients with treatment success according to Feres et al. (2020) at T1 (**a**) and T2 (**b**) regarding both BOP and residual pocket depths

Analysing treatment success according to Feres et al. (2020) at T1 revealed that a defined endpoint of BOP < 20% was achieved in 49% of all patients, and the endpoint of ≤ 4 sites with PD ≥ 5 mm in 29% of all patients (Fig. 4a). At T2, the endpoint of BOP dropped to 37% and to 26% in terms of PD (Fig. 4b).

### Factors influencing T1 and T2 endpoint achievements

After Step 2 of periodontal therapy (T1), the BOP endpoint was positively associated with female sex (*p* = 0.017) and negatively associated with PCRT1 (*p* = 0.027) and BOPT0 (*p* < 0.001) indicating a negative influence of inflammation at T0 and plaque at T1. After adjusting for age and sex, PCR lost significance, while BOPT0 remained significant (*p* < 0.001), including in the fully adjusted model.

Achieving the PD endpoint at T1 was negatively influenced by smoking (*p* = 0.041), BOPT1 (*p* = 0.012) and the number of sites with PD ≥ 6 mm at T0 (*p* < 0.001), highlighting the negative roles of smoking, deep pockets, and persistent periodontal bleeding.

After adjustment, BOPT1 and smoking lost significance, while PD ≥ 6 mm at T0 remained significant (*p* < 0.001).

At T2, BOP success was positively linked to female sex (*p* = 0.018), PCRT2 (*p* < 0.001), BOPT1 (*p* = 0.004), and SPC adherence (*p* < 0.005), showing that female sex and adherence support BOP success while plaque and bleeding impair it.

In the multivariate analysis, only SPC adherence and female sex remained significant predictors for BOP < 20%. At T2, PD success was negatively influenced only by sites with PD ≥ 6 mm after Step 2, which remained significant across models, marking these sites as predictors for lower PD success.

Full univariate and multivariate results are shown in Table 3.

**Table 3:** OR with 95% CI and p-values from nominal regression analysis for different variables associated with clinical treatment outcomes at T1 and T2, according to Feres et al. (2020)

dependent variable	variable	Univariate				Model 1				Model 2			
		OR	L-CI	U-CI	*p*-value	OR	L-CI	U-CI	*p*-value	OR	L-CI	U-CI	*p*-value
T1 BOP endpoint	female	2.58	1.18	5.64	0.017*	3.89	1.45	10.45	0.007*	3.90	1.35	11.25	0.008*
	PCR (T1)	0.14	0.02	0.80	0.027*	1.00	0.97	1.02	0.732	0.99	0.97	1.02	0.665
	BOP (T0)	0.02	0.00	0.12	< 0.001*	0.95	0.93	0.97	< 0.001*	0.95	0.92	0.98	< 0.001*
T1 PD endpoint	BOP (T1)	0.97	0.94	0.99	0.012*	0.86	0.79	0.94	0.275	0.88	0.80	0.96	0.315
	PD ≥ 6 mm (n) (T0)	0.85	0.78	0.92	< 0.001*	0.98	0.95	1.01	< 0.001*	0.98	0.95	1.02	< 0.001*
	smoking	0.33	0.11	0.96	0.041*	0.33	0.10	1.17	0.086	0.28	0.06	1.19	0.848
T2 BOP endpoint	female	2.63	1.17	5.89	0.018*	1.60	0.60	4.26	0.351	1.42	0.48	4.20	0.528
	PCR (T2)	0.95	0.92	0.97	< 0.001*	0.96	0.93	0.98	0.002*	0.96	0.93	0.99	0.003*
	BOP (T1)	0.97	0.94	0.99	0.004*	0.98	0.95	1.01	0.294	0.98	0.94	1.01	0.150
	SPC-NA	1.90	1.30	2.79	< 0.001*	1.48	0.91	2.40	0.111	1.53	0.85	2.76	0.158
T2 PD endpoint	PD ≥ 6 mm (n) (T1)	0.84	0.73	0.96	0.010*	0.84	0.73	0.96	< 0.001*	0.69	0.55	0.87	< 0.001*

Abbreviations and notes: OR = odds ratio, L-CI = lower confidence interval, U-CI = upper confidence interval, PD = pocket depth, BOP = bleeding on probing, PCR = plaque control record, SPC-NA = conducted supportive periodontal care (SPC)/year and the recommended SPC/year; * statistically significant

## Discussion

Non-surgical periodontal therapy significantly improved all clinical parameters from T0 to T1; *p* < 0.001). However, during SPC (T1-T2), these gains declined, with plaque control worsening significantly (*p* < 0.001). Achieving a BOP ≤ 20% was met by 49% of all patients at T1, positively influenced by adherence to SPC intervals, female sex, and lower baseline PCR/BOP. In contrast, PD target success (≤ 4 sites with PD ≥ 5 mm) was met by only 29% of all patients at T1, negatively effected by baseline PD ≥ 6 mm. Smoking was a significant negative factor in univariate analyses, however it revealed as not to be an independent factor in multivariate analysis. This study systematically identified factors influencing treatment success and RPSTN periodontal care performed by students, supporting prior findings on inflammation reduction [35–38]. Particulary persistent deep pockets highlight the need for advanced strategies beyond non-surgical approaches, especially in complex cases [16].

### Deep residual pockets after Step 2 and Factors influencing BOP ≤ 20%

Residual pockets with PD ≥ 5 mm and even more with PD ≥ 6 mm are a known risk factor for disease progression, even after non-surgical therapy, as outlined in current guidelines [16, 23, 24, 50, 51]. However, not all such pockets require immediate retreatment. Still, PD ≥ 6 mm - even without BOP - is linked to increased long-term risk (Matuliene et al., 2008) and is considered a clinical marker for surgical need. This analysis does not suggest overtreatment but highlights the prevalence and predictors of these residual sites in periodontal therapy performed by students. Beside the Feres endpoint for PD, in this cohort, 80% of patients had at least one site requiring surgery at T1, persisting in 79% at T2. These findings underscore not only the limitations of non-surgical therapy but also the substantial surgical treatment need. Smoking showed univariate significance on treatment success in terms of PD but lost predictive value after adjustment, likely due to missing data on daily cigarette consumption, which affects outcomes dose-dependently [52].

Achieving BOP ≤ 20% post-Step 2 was primarily driven by effective plaque control (PCR reduction: *p* < 0.001), with the female sex showing a significant positive association. By T2, adherence to risk orientated SPC intervals was sole independent predictor (*p* = 0.003), underscoring its supremacy over other factors like SPC adherence or baseline BOP, which lost significance after adjustment.

### Practical implications arising directly from this study’s findings

#### Plaque control as a core curriculum component

While systemic inflammation modulation (e.g. via diet [53]) may support outcomes, PCR’s importance highlights the need for practical plaque control training.

Communication methods like motivational interviewing [54, 55] could be included to develop patient dialogue skills, though long-term effectiveness remains controversial [56]. Given BOP’s relevance, lifestyle factors affecting immunity and plaque environment should be part of multimodal treatment concepts [57, 58], though further research is needed.

#### Structural reforms for SPC adherence

The gap between risk-based recall intervals and attendance [17] likely results from rigid semester schedules limiting access for high-risk patient.

Longitudinal patient-student assignments [59] and integrated courses (per German licensing reforms) could enhance continuity and support surgical training, as outlined in the *Graduated Clinical Training* section.

#### Strategic use of adjunctive therapies

Microbiological tests were strongly promoted in the past, and the main observation period of this study still reflects those recommendations [48, 60, 61]. However, such tests are now outdated, as they detect only a few bacteria and the role of many others remains unclear [62]. Their use in undergraduate education can therefore be omitted. Systemic antibiotics (amoxicillin/metronidazole) reduce residual pockets in severe cases [63, 64]. In patients with deep periodontal pockets (PD > 5 mm) who are younger than 55 years [64], and particularly in those under 35 years of age [62, 65], adjunctive antibiotic therapy has been shown to result in significant and clinically relevant site-specific reductions. Therefore, while the use of antibiotics should always be critically evaluated, it is clearly indicated in cases of severe or rapidly progressing disease and is not rejected in current studies or clinical guidelines [16, 62].

The limited effect of antibiotics in periodontal therapy may be attributed to several factors. Historically, prescribing practices were influenced by microbial testing prior to the introduction of standardized guidelines, which may have restricted antibiotic use [48]. may have restricted antibiotic use. Additionally, patient scepticism regarding antibiotics and growing concerns about their overuse [66–69], combined with issues of adherence to prescribed regimens [70, 71], may have further compromised their overall effectiveness. For these reasons, both students and instructors should be trained in the guideline-based use of antibiotics, and patient communication should be improved and strengthened through dedicated seminars to help overcome patient scepticism. Nevertheless, students must also be thoroughly instructed in the cautious use of antibiotics, particularly with regard to the risk of developing antimicrobial resistance [40, 62].

### Extended implicit guidance for curricular refinement

#### Graduated clinical training

The high prevalence of residual PD ≥ 6 mm (at T1, 80% of all patients; at T2, 79%) raises questions about training models. While limiting students to “simple” cases (PD ≤ 5 mm) may leave them unprepared for clinical realities in practice, exposing them to complex cases without structured guidance can compromise results. This issue is also highlighted in the findings from DMS 6 [30]. In the future, an increasing number of older patients will retain more teeth, many of which are likely to present with deeper periodontal pockets (PD > 5 mm), which—according to current guideline recommendations—should preferably be treated surgically. Given the currently limited number of specialists available within reasonable proximity to dental practices in Germany, this may become problematic if inexperienced general practitioners either perform surgical interventions without sufficient training or deliberately avoid surgery. Therefore, offering low-threshold surgical training already at the undergraduate level would be a meaningful step.

A graded competency framework could offer a solution:

- Early semesters: Treat mild-moderate cases (PD ≤ 5 mm) to build non-surgical skills.
- Advanced semesters: Manage complex cases (PD ≥ 6 mm) with close supervision and surgical integration by direct assistance at surgical measures, aligning with the EFP S3 guidelines [16] and current German licensing reforms [27].

This concept would also reflect the findings of DMS 6, which showed that although the average number of teeth among seniors increased, the mean probing depth also rose again—particularly in severe cases with a probing depth of ≥ 6 mm [30].

#### Lifestyle interventions as part of training

Lifestyle factors (e.g. smoking, sleep quality and diet) are increasingly recognized as key to treatment success [72, 73]. The importance of both understanding and teaching this topic was also emphasized during the 19th Workshop on Periodontology [40]. Including these in Step 1 serves dual purposes:

- Student learning: Prepares students to manage periodontitis as a multifactorial disease, linking systemic and clinical knowledge.
- Patient benefits: Promotes better outcomes through counselling on risk factor control like smoking and stress management, complementing mechanical therapy.

#### Surgical integration in undergraduate training

Given that at T2 still a median of 2% of sites showed a PD ≥ 6 mm and presence of those pockets at T0 and T1 significantly affected the treatment outcomes in terms of PD, surgical intervention should be increasingly considered, and students should have acquired the necessary knowledge by graduation. Ideally, they should have participated and assisted with surgical periodontal procedures multiple times to build their surgical competence. The revised German licensing framework [27] supports interdisciplinary collaboration with oral surgery departments for hands-on training in flap surgery/pocket reduction using simulation units and e.g. pig jaws. Structured modules could include simulated training, assisting in surgery, and possibly performing supervised measures (e.g. placing simple final suturs), similar to supervised tooth extractions. This approach addresses limitations in smaller university departments (e.g. limited chairs) while ensuring graduates meet EFP S3 standards [16]. Naturally, postgraduate periodontal training—particularly the acquisition of technically demanding and often costly treatment techniques such as free connective tissue grafts from the palate, aesthetic soft tissue periodontal surgery, the use of bone substitute materials, or membrane techniques—remains unaffected by this [74].

### Limitations and strengths

The following limitations should be stated.

1. *Smoking behaviour*: The lack of detailed documentation on smoking intensity (e.g. pack-years and daily cigarettes counts, and cessation timelines) limited the ability to analyse dose-dependent effects, which are crucial given the well-established link between smoking and periodontal progression [75–77]. At that time, only the smoking information required to create the periodontal risk diagram per Lang and Tonetti was requested and recorded [17]. However, in contrast to what is specified by the classification, no differentiation was made among active smokers based on the number of cigarettes consumed per day. Instead, all smokers were assigned to Grade B, although a consumption of ≥ 10 cig./d would have more appropriately warranted classification as Grade C. As a result, the number of patients categorized as Grade C may have been underestimated in favor of those classified as Grade B.
2. *Metabolic parameters*: HbA1c values were unavailable for diabetic patients, preventing analysis of the effect of glycaemic control on treatment outcomes. This limitation also affected retrospective re-grading accuracy, possibly leading to an underestimating of the disease grade, particularly in patients with undiagnosed or uncontrolled diabetes.
3. *Sample size*: The small number of high-risk patients (e.g. *n* = 14 diabetics, *n* = 33 smokers) likely reduced the statistical power of subgroup analyses, increasing the risk of Type II errors for variables such as antibiotic use. This restricts the generalisability of findings to clinical populations with more severe systemic risk profiles.
4. *Retrospective design and examiner variability*: Periodontal assessments were carried out by different individuals (students and supervisors) which may have introduced inter-examiner variability in PD/BOP measurement, despite standardised training and protocols. Likewise, treatment outcomes could be influenced by differing skill levels between novice and advanced students, as well as varying degrees of supervisor intervention. However, this reflects the variability of real-world educational settings. It cannot be ruled out that, despite years of use and familiarity with the periodontal probe PCB 11, diagnostic reading errors may have occurred in some cases by individual students. This could represent a potential, albeit minor, source of bias.
5. *Diagnosis reclassification*: The retrospective application of the 2018 Periodontal Classification System followed common practice in cohort studies and was based on existing clinical and radiographic records. Still, reclassification may introduce bias, especially regarding the accurate reflection of disease severity.
6. While the treat-to-target endpoint was originally validated for 12-month outcomes, the present study applied it after a median follow-up of 47 months. This extended interval may have introduced reinfection or disease progression, thereby limiting the comparability to short-term controlled settings.

**Strengths** of this study include the availability of complete periodontal documentation (PD, BOP, PCR), which supports strong internal validity. Furthermore, the setting reflects authentic conditions of undergraduate training, where variability in practitioner experience represents the natural transition from simulation to clinical reality.

**Generalisability** may be somewhat limited, as findings stem from an academic environment rather than private practice, where more experienced clinicians treat patients. On the other hand, the absence of surgical procedures may realistically reflect the scope of care provided by general dentists in Germany and comparable healthcare systems [50]. Additionally, the demographic profile of the cohort (median age 58yrs) may not fully capture populations with more complex systemic comorbidities.

**Future directions** should include prospective studies with examiner calibration, continuous monitoring of smoking behaviour and glycaemic control, and larger cohorts of high-risk patients. Incorporating patient-reported outcomes, such as barriers to treatment adherence, could provide valuable context for understanding the educational and clinical challenges.

Two key aspects are worth highlighting:

During the observation period (2004–2018), the applied non-surgical treatment protocol was partly based on the findings of Kaldahl et al., who reported minimal long-term differences between non-surgical therapy and the modified Widman flap procedure—even at sites with PD ≥ 7 mm [78]. The reported mean difference to osseous surgery was only 0.5 mm.

Many patients in this study expressed satisfaction after Step 2, reporting improved periodontal conditions such as reduced bleeding and less unpleasant taste. As a result, numerous patients declined additional interventions like antibiotics or surgical procedures.

### Summary of recommendations for education, treatment and future research

Based on this study’s findings and important findings from recent studies on factors that affect periodontal inflammation beyond the sole presence of plaque, the following dental curriculum reforms are proposed:

1. **Integrated nutrition and lifestyle education**: Expand training on lifestyle factors (e.g. smoking cessation, diet, stress) and their impact on periodontitis, supported by structured communication seminars for patient counselling (see *Factors Influencing BOP ≤ 20%*);
2. **Longitudinal clinical training**: Relax rigid semester structures to allow continuous patient care across terms, supporting adherence to risk-based SPC or grading-related intervals, as practiced in Germany. Assigning patients to students for the full clinical may further improve continuity [59];
3. **Treatment protocols**: Emphasising the guideline-compliant use of antibiotics (amoxicillin/metronidazole) to reduce PD and thus reduce surgical need (see *Adjunctive Therapies*). Following intensive and comprehensive interdisciplinary training on dental simulators and pig jaws, students may assist in selected complex cases (PD ≥ 6 mm) by participating directly in surgical procedures. Under supervision, they also have the opportunity to perform specific tasks independently—such as placing a simple final suture—in line with the S3 guidelines of the EFP and the current reforms of the German dental licensing regulations (see *Surgical Integration*).

**Future research** should assess the effects of continuous patient assignments and lifestyle counselling on clinical outcomes, as well as evaluate the benefits of integrated surgical training for reducing RPSTN rates and improving student competency.

## Conclusion

This study shows that subgingival instrumentation within the scope of non-surgical periodontal treatment performed by students can lead to measurable improvements in clinical parameters among patientes with stage III periodontitis and residual pockets ≥ 6 mm represent a fundamental problem and should be addressed through appropriate measures within the scope of student treatment. However, several aspects emerge that could influence an improvement in outcomes.

Both more effective plaque control and adherence to risk-based SPC intervals—especially when combined with efficient plaque management—would likely have a stronger influence on treatment outcomes and the attainment of stricter therapeutic goals.—such as the treat-to-target criteria. Notably, however, the persistence of residual pockets continues to have a significant impact on treatment outcomes and the aforementioned therapeutic targets in many cases. This includes, among other things, the indication-based use of antibiotics for certain patient groups, the integration of current scientific findings into treatment protocols, and increased surgical intervention, in which students should actively participate.

### Transferred implications of the conclusions

Although this study did not directly assess educational interventions, the observed treatment limitations point to several curricular aspects that may warrant further investigation:

1. Graduated clinical training, where advanced students manage complex cases (PD ≥ 6 mm) according to current guidelines.
2. Longitudinal patient-student assignments to ensure continuity of care and adherence to risk-adjusted SPC intervals.

### Implicit recommendations from the conclusions

Based on increasing awareness regarding the bidrirectional relationship between systemic and periodontal inflammation and its relationship to certain lifestylefactors and based on the fact that such topics were not part of the educational curriculum within the investigated cohort, we suggest to systematically integrate lifestyle counselling training to empower students to address multifactorial disease drivers (e.g. smoking, plaque control, nutrition, body weight) and thereby address shared risk factors for other diseases.

Future research should evaluate the impact of these strategies on clinical outcomes while addressing systemic barriers (e.g. patient compliance with antibiotics and surgical training scalability). As care performed by students expands, such evidence-based adaptations will be pivotal in aligning educational objectives with modern periodontal standards.

## Electronic supplementary material

Below is the link to the electronic supplementary material.

Supplementary Material 1

## Data Availability

The datasets used and/or analysed during the current study are available from the corresponding author on reasonable request.
