# Supplementary material for: Student-performed periodontal therapy: retrospective cohort study on outcomes and related recommendations for enhancing undergraduate periodontal education
Source: BMC Med Educ. 2025 Jul 31;25:1130. doi: 10.1186/s12909-025-07699-2 (PMC12312536; doi:10.1186/s12909-025-07699-2)
Supplement: Supplementary file 1 — Supplementary Material 1 [file 12909_2025_7699_MOESM1_ESM.docx]

**Appendix 1: Procedure for standardized periodontitis treatment at the dental clinic**

**Step 1**: At PER 1 (pre-periodontal treatment session 1), the patient’s medical history was recorded. All periodontitis patients followed a consistent treatment protocol. The aetiology and treatment concept were explained, followed by instructions for improved oral. This included low-abrasive fluoride toothpaste, an oscillating-rotating or sonic electric toothbrush (Oral-B, Procter & Gamble Service GmbH, Schwalbach am Taunus, Sonicare™, Philips GmbH Market DACH, Hamburg, Germany), appropriate interdental brushes, and a 14-day application of a 0.2% chlorhexidine (CHX) mouth rinse (Meridol® med CHX 0.2%, CP GABA GmbH). Oral hygiene was assessed the plaque control record (PCR) by O'Leary et al. and the papilla bleeding index (PBI) by Saxer and Mühlemann (O'Leary *et al.*, 1972; Saxer & Mühlemann, 1975). New or recent (≤6 months) radiographs of all teeth were collected for diagnostics and insurance compliance. Supragingival plaque and biofilm were removed using hand and ultrasonic scalers, followed by polishing (Cleanic™ Prophy-Paste, Kerr™ GmbH)., At PER 2 (after 14 days), compliance was checked using PCR and PBI, and patients were re-instructed. Periodontal diagnosis included PD, BOP, FI and TM, and followed by another supragingival cleaning. Measurements were taken using the PCP11 periodontal probe and Naber PQ2N probe (Hu-Friedy Mfg. Co., LLC).

**Step 2**: In the oral hygiene phase, a modified full-mouth disinfection protocol (Quirynen *et al.*, 1995) was used. Patients rinsed with a 0.2% CHX and brushed the tongue with 1% CHX gel for 1 minute pre-treatment. SRD 1 (scaling and root-debridement session 1) was performed under infiltrative or intraligamentary anaesthesia depending on the patient’s preferences (Ultracain; Sanofi-Aventis Deutschland GmbH). Subgingival instrumentation was carried out for all PD≥4 mm using Gracey curettes (Gracey, Hu-Friedy Mfg. Co., LLC.) and sonic scalers (SONICflex™, KaVo Dental GmbH). If > 14 teeth required treatment, SRD 2 was done the next day. After cleaning, 1% CHX gel was applied to pockets, and patients rinsed with 0.2% CHX for 14 days. After 6-8 weeks, patients were re-evaluated. If PD≧4mm with BOP persisted, retreatment occurred in the same session.

**Step 3**: No surgical procedures were performed. Instead, this step was replaced with an additional non-surgical session, as described in Step 2.

**Step 4**: Following debridement, a periodontal risk assessment (PRA) per Lang and Tonetti (2003) was conducted to determine recall intervals. Patients were then transferred to supportive periodontal care (SPC). During the SPC visits, PER 2 content was repeated, new periodontal status recorded and teeth with PD≥4mm and BOP were re-instrumented subgingivally using curettes or ultra-/sonic scalers. Figure 2 provides a graphical representation of the standardized protocol for periodontal therapy.
